# Alcohol- and Cigarette-Use Related Behaviors During Quarantine and Physical Distancing Amid COVID-19 in Indonesia

**DOI:** 10.3389/fpsyt.2021.622917

**Published:** 2021-02-02

**Authors:** Enjeline Hanafi, Kristiana Siste, Albert Prabowo Limawan, Lee Thung Sen, Hans Christian, Belinda Julivia Murtani, Adrian, Levina Putri Siswidiani, Christiany Suwartono

**Affiliations:** ^1^Department of Psychiatry, Faculty of Medicine Universitas Indonesia, Cipto Mangunkusumo Hospital, Jakarta, Indonesia; ^2^Atma Jaya Catholic University of Indonesia, Jakarta, Indonesia

**Keywords:** physical distancing, large-scale social restriction, alcohol, cigarette, prevalence

## Abstract

**Background:** In light of the coronavirus disease 2019 (COVID-19) pandemic, Indonesia implemented large-scale social restrictions (*pembatasan sosial berskala besar*/PSBB) to combat the spread of COVID-19, which might influence addictive behaviors. The current study aimed to explore the fluctuation of substance use during the pandemic and association of physical distancing and related factors toward consumption of alcohol and cigarettes.

**Method:** An online survey was conducted from April 28 to June 1, 2020. Data regarding sociodemographic information, physical distancing profile, alcohol and cigarette usages, Alcohol Use Disorders Identification Test (AUDIT), Cigarette Dependence Scale (CDS), Symptom Checklist-90, and Pittsburg Sleep Quality Index (PSQI) were collected. A total of 4,584 respondents from all 34 provinces in Indonesia completed the survey. Data were summarized descriptively and analyzed using chi-square, ANOVA, and multinomial regression on SPSS 23.0 for Windows.

**Results:** This study found that during the COVID-19 pandemic in Indonesia alcohol consumption was 9.50% and daily cigarette smoking was 20.3%. Around 44.5% and 47.6% of respondents reported stable alcohol consumption and cigarette consumption, respectively. The mean AUDIT score was 3.52 ± 4.66 and the mean CDS score was 24.73 ± 8.86. Physical distancing was not correlated to any substance use changes. Increased alcohol consumption was negatively correlated with being unmarried and positively correlated with a higher PSQI score. Decreased alcohol use positively correlated with living in PSBB-implementing provinces and higher AUDIT scores when compared to stable alcohol drinking. Increased cigarette smoking was positively correlated with being male, unmarried, and higher CDS scores. Reduced cigarette smoking was negatively correlated with living in provinces implementing PSBB, higher CDS scores, and phobic anxiety, hostility, and psychoticism subscales of SCL-90.

**Discussion and Conclusion:** The prevalence of alcohol and cigarette consumption changes showed a similar trend with other available studies in other countries. This study established that substance use was mainly sustained with a smaller proportion of respondents amplifying their substance usages. The changes were correlated with PSBB policy but not the practice of physical distancing. Psychiatry and addiction services in Indonesia should be strengthened to cope with the increased burden of psychological distress. Future studies should conduct more comparisons to determine whether the overall rising intensity of consumption was maintained post-pandemic and delineate acute psychopathologies' effects on substance use.

## Introduction

On March 11, 2020, the World Health Organization (WHO) declared a pandemic of a novel coronavirus, known as severe acute respiratory syndrome coronavirus 2 (SARS-CoV-2). The spread and severity of this condition continues to impact the world to date. World Health Organization has reported more than 23 million confirmed cases and 800,000 confirmed deaths in 216 countries (1). Indonesia, the fourth most populous country in the world, reported more than 150,000 confirmed cases and 6,500 deaths due to the coronavirus disease 2019 (COVID-19) as of late August 2020 (2, 3).

In response to the pandemic, the Indonesian government announced the implementation of large-scale social restrictions (*pembatasan sosial berskala besar*/PSBB) to accelerate COVID-19 eradication. In the PSBB, schools, workplaces, and public places were closed, and mass transport was reduced. Activities involving large gatherings, including those for religious purposes, were restricted, and people were advised to stay at home (4). According to a study conducted by the Indonesian Psychiatrist Association from April to August 2020, which included 4,010 subjects (aged 17–29 and over 60 years), ~64.8% of the respondents experienced at least one psychological problem. Among the respondents with psychological problems, almost 65% experienced anxiety, 61.5% had depressive symptoms, and 74.8% reported post-traumatic complaints during the pandemic (5). Social (and physical) distancing and quarantine or isolation was meant to prevent further COVID-19 transmission; however, it could lead to the worsening of several negative psychological symptoms (6). In some individuals, this could also lead to unfavorable behavior such as substance abuse in order to relieve symptoms (7). A previous study found a relationship between the SARS outbreak in Beijing in 2003 and alcohol abuse/dependence symptoms 3 years later among hospital employees and described one of the risk factors as being a history of quarantine (8). Changes in substance use levels might vary as increased consumption is possible due to heightened emotional distress, isolation, and unemployment, and a decrease in its consumption is possible due to reduced availability, higher prices, and financial restrictions.

In 2018, The Indonesian Basic Health Research showed that the prevalence of alcohol drinking among Indonesians older than 10 years old during the past year was 3% (9). Furthermore, the prevalence of heavy episodic drinking (consumption of at least 60 grams or more of pure alcohol on at least one occasion in the past 30 days) during the past year among Indonesians older than 15 years was 6.5% in 2016. It was reported that the overall prevalence of alcohol use disorders was 0.8%, while alcohol dependence was 0.7% (10). These rates are lower than the WHO South-East Asia Region's prevalence of 3.9% for alcohol use disorders and 2.9% for alcohol dependence (10). The Indonesian 2018 Basic Health Research stated that the prevalence of past-year tobacco consumption in those aged above 15 years was 33.8% (11), where the prevalence of daily tobacco smoking was 24.3%, and that of e-cigarette use was about 2.8% (12). Considering the already high prevalence of substance use among Indonesians prior to the pandemic and the distress caused during PSBB, it is essential to determine substance (alcohol and cigarette) consumption changes. The study aimed to explore in detail the fluctuation in usage of substances, particularly of alcohol and cigarettes, during the pandemic. We hypothesized that the pandemic and PSBB affected alcohol and cigarette consumption behavior. Complementarily, this study would also explore the effect of physical distancing and other factors, including psychopathologies and sleep disturbance, during this pandemic on the use of alcohol and cigarettes. The results of this study would be beneficial for the development of evidence-based strategies for the management of substance use post-COVID-19 or in the new normal period.

## Methods

### Respondents and Procedure

The questionnaire was opened from April 28 to June 1, 2020 employing an online survey platform, *Google Form*. Online data collection was initiated about 42 days after the declaration of PSBB. The research team disseminated the link address for the online survey through several social media platforms. Furthermore, the online survey link was shared with Indonesian state-owned companies, university lecturers and students, and respondents, who were encouraged to disseminate the link for this online survey.

Before participating in the survey, respondents were asked to provide informed consent after the study purpose, respondent criteria, and data management were presented to them. Email for correspondence was provided for any inquiries. This online survey comprised a demographic section (e.g., age, gender, formal education, occupation, current residency, marriage, and household income), substance use consumption detail [alcohol and daily cigarette consumptions since the start of the COVID-19 pandemic (first reported case of COVID-19 in Indonesia was on March 2, 2020), their perceived change of current use compared to before the pandemic (unchanged, increased, or decreased), and the option of ‘do not use’ denoted have not used ever in life], physical distancing profile (practice and location), the Alcohol Use Disorders Identification Test (AUDIT), Cigarette Dependence Scale (CDS), Symptom Checklist 90 (SCL-90), and Pittsburg Sleep Quality Index (PSQI). Each questionnaire item was set as mandatory; thus, respondents with missing data were unable to submit. However, *Google Form* did not allow for calculation of the response rate as it did not record the total number of surveys accessed. In this study, physical distancing was defined as studying or working from home, alternating school or working days, and/or the practice recommended by the Indonesian COVID-19 Response Acceleration Task Force. Current residency information was collected based on provinces and further categorized based on status of PSBB implementation in that province according to the *Indonesian National Board for Disaster Management* by April 28 2020, which consisted of DKI Jakarta, West Java, East Java, Central Java, Banten, West Kalimantan, North Kalimantan, Gorontalo, West Sumatera, Riau, and South Sulawesi. Household income was classified based on gross national income classification by the World Bank.

This study's inclusion criteria were that respondents were aged above 21 years, currently residing in Indonesia, and able to understand Indonesian. Several responses, which were non-consenting (*n* = 23), duplicates (*n* = 5), and currently not residing in Indonesia (*n* = 13), were excluded. The email addresses of the respondents were collected to prevent multiple responses from an individual, and they were only accessible to the research team and were removed after the elimination of duplicate responses. Overall, there were 4,584 responses (56.1% males) with respondents from all 34 provinces and 7 islands (Java 62.7%, Sumatera 18.3%, Borneo 8.6%, Sulawesi 5.8%, Nusa Tenggara 2.7%, Papua 1.7%, and Maluku 0.3%) across Indonesia.

### Instruments

#### The Alcohol Use Disorders Identification Test

This questionnaire was developed as a screening instrument to identify the effects of dependence and harmful use of alcohol, designed to be used in primary health care, and was the only alcohol screening test applicable for international use. This questionnaire comprises 10 questions focusing on the recent use of alcohol; scoring ranges from 0 to 40 with a score 8–14 interpreted as harmful alcohol use and ≥15 as a possibility for dependence (13). The WHO collaborative study showed that the AUDIT is a valid instrument in six countries (sensitivity 92% and specificity 94%) (14). In this study, the AUDIT demonstrated acceptable internal consistency (α = 0.80) among alcohol drinkers.

#### Cigarette Dependence Scale-10

This self-reported questionnaire aids in determining the severity of nicotine dependence (15). Each question has five multiple-choice answers. Question number 1 asked cigarette dependency with scoring 0 to 100 being divided into five intervals (0–20, 21–40, etc.). Question number 2 asked the number of cigarettes smoked which ranges from 0 to more than 30 rolls divided into five intervals (0–5, 6–10, etc.). Question number 3 used Likert scale with values from 1 to 5, as “very easy” to “impossible.” Meanwhile, the Likert Scale used in the rest of the questions were as “completely disagree” to “highly agree.” The output of this questionnaire is in a numeric form with no determined cutoff number, and a higher score indicates more severe nicotine dependence. Evaluation of the Indonesian version of CDS showed that a modification of the CDS from 12 to 10 questions improved the instrument's statistical value with good reliability (Cronbach's alpha = 0.91, ICC = 0.91) (16). The excluded items were question number 3 (first cigarette of the day) and 9 (too much smoking). Thus, CDS-10 was used in this study. In this study, the CDS demonstrated acceptable internal consistency (α = 0.92) among cigarette smokers.

#### Symptom Checklist-90

The self-reported questionnaire comprises 90 statements scored on a 5-point Likert scale, 0 (=Never) to 4 (=Always), within the last 30 days. The Indonesian version of the SCL-90 showed good validity, 82.9% sensitivity, and 83.0% specificity (17). This questionnaire is used to assess psychopathological symptoms, including somatization (distress concerning physical problems), obsessive-compulsive (relating to irresistible, repetitive, and unwanted impulses, thoughts, and actions), interpersonal sensitivity (negative expectations, self-doubt, and feeling inferior in a relationship with other people), depression (dysphoria, loss of pleasure, pessimism, etc.), anxiety (nervousness, apprehension, dread, and trembling), hostility (aggression, irritability, and rage), phobic anxiety (irrational or excessive fear relating to persons, places, objects or situations), paranoid ideation (thought of hostility, grandiosity, and suspiciousness and need for control based on fear), psychoticism (extremely isolated and core symptoms of schizophrenia, including hallucination and thought control), an additional subscale (poor appetite, sleep disturbance, fear of dying, and overeating), and an overall global symptom index (GSI) (18–20). In this study, SCL-90 demonstrated acceptable internal consistency, ranging from α= 0.84 to 0.93 across the 10 domains and α = 0.99 for the GSI, among alcohol and cigarette users.

#### Pittsburgh Sleep Quality Index

The PSQI is a commonly used instrument in assessing sleep quality in clinical or non-clinical populations (21, 22). The questionnaire is comprised of 24 items, of which 20 are multiple choice questions and four are open-ended questions. Furthermore, five items required the assessment of a partner or another individual on the respondent's sleeping pattern. The 19 self-answered questions on PSQI can be pooled into seven components and each weighted between 0–3 (maximum 21), scores >5 indicate poor sleep quality. The Indonesian version of PSQI was validated with a reliability of α = 0.79, content validity 0.89, and specificity of 81% (23). In this study, PSQI demonstrated acceptable internal consistency (α = 0.83) among alcohol and cigarette users.

### Data Analysis

Descriptive analyses were performed for all data; demographic data were presented against substance consumption characteristics during the COVID-19 pandemic. The association between sociodemographic factors and substance consumption characteristics was generated by Chi-square. Psychometric data were analyzed using One-way ANOVA, and significant groups were further tested using Tukey's or Games-Howell *post-hoc* analysis depending on the Levene's-test of equal variance results. Finally, all variables were scrutinized simultaneously using multinomial regression analysis with unchanged substance consumption set as the reference category. All statistical tests were performed using SPSS 23.0 for Windows (IBM, USA). Data were deemed significant if *p* < 0.05.

### Ethics and Approval

This study was approved by the Institutional Ethics Committee of the Faculty of Medicine, Universitas Indonesia—Dr. Cipto Mangunkusumo General Hospital (KET-413/UN2.F1/ETIK/PPM/00/02/2020). All respondents provided written informed consent.

## Results

### Prevalence and Sociodemographic of Alcohol and Cigarette Usages

The prevalence of the consumption of alcohol and daily cigarette smoking during the COVID-19 pandemic found in this study was 9.50% (*N* = 436) and 20.31% (*N* = 931). Regarding alcohol use, 44.5% reported no change in usage, while 29.8% reported reduced usage, and 25.7% reported increased usage. The data for cigarette smoking showed that 47.6% reported maintained usage, 32.3% reported reduced usage, and 20.1% reported increased usage.

Among alcohol drinkers (*N*= 436), the mean age was 30.4 ± 6.8 with 60.3% being male, 43.3% unmarried, and 43.8% lived in PSBB-implementing provinces. Married alcohol drinkers reported a significantly larger proportion of increased drinking (40.2%) than unchanged (35.4%) or reduced (24.3%) alcohol consumption. Those living in provinces implementing PSBB reported a higher proportion of decreased (35.6%) drinking than increased (22.0%). Among smokers (*N* = 931), the mean age was 33.3 ± 7.3 with 93.5% being male, 73.9% unmarried, and 41.7% lived in provinces implementing PSBB. Most male smokers either maintained, 48.4%, or decreased, 32.9%, their cigarette consumption. A significantly higher proportion, about 49.4%, of unmarried smokers recounted an unchanged number of cigarettes smoked, and only around 18.0% reported increased smoking. The data are shown in Supplementary Table 1.

### Descriptive Psychometric Data

Respondents disclosing increased alcohol consumption tended to be significantly older than those who reported stable and decreased drinking [*F*_(2, 433)_ = 10.16, *p* ≤ 0.001]. The mean AUDIT score was 3.52 ± 4.66 and, categorically, 10.1% respondents reported harmful alcohol use and 4.4% reported alcohol dependence. Those with reduced alcohol consumption had significantly higher AUDIT scores than those with stagnant and increased alcohol consumptions [*F*_(2, 433)_ = 7.99, *p* ≤ 0.001]. The mean CDS-10 score was 24.73 ± 8.86. This study demonstrated a significant difference of CDS-10 scores among smoking pattern changes [*F*_(2, 928)_ = 35.72, *p* ≤ 0.001]. Respondents that reported an increase in cigarette smoking scored 28.72 ± 9.03 which was significantly higher than the scores of those with constant smoking (24.89 ± 8.863) and those that disclosed reduced cigarette consumption (22.01 ± 7.720). Of the 436 alcohol drinkers and 931 cigarette smokers, 45.9 and 43.8% had poor sleep (PSQI score > 5), consecutively. PSQI scores did not significantly differ among changes in the use of both substances (shown in Supplementary Table 2).

### Overall Correlates of Substance Consumption Changes

The multinomial regression data are shown in Table 1. The perceived stable consumption pattern was used as the reference category. Living in provinces implementing PSBB (aRRR = 2.14, *p* = 0.008) and higher AUDIT scores (aRRR = 1.11, *p* ≤ 0.001) were positively correlated with decreased alcohol consumption, when compared to those who reported stable alcohol use. Attaining a university degree (aRRR = 2.38, *p* = 0.045) and higher PSQI scores (aRRR = 1.11, *p* ≤ 0.04) were correlated with higher risk of increased rather than stable alcohol consumption; while those who were single were less likely to report increased alcohol use (aRRR = 0.31, *p* ≤ 0.001). Male respondents (aRRR = 2.70, *p* = 0.006) and those who were single (aRRR = 1.69, *p* = 0.03) were positively correlated with increased cigarette consumption than those with stagnant smoking. Living within PSBB-implementing provinces (aRRR = 0.68, *p* = 0.03) was linked with lower odds of decreased smoking than stagnant cigarette consumption. Higher CDS score was more likely to predict increased than stagnant cigarette consumptions (aRRR = 1.06, *p* ≤ 0.001) but less likely for decreased smoking (aRRR = 0.95, *p* ≤ 0.001) compared to stable cigarette use. Those with higher scores in phobic anxiety (aRRR = 0.70, *p* = 0.03), hostility (aRRR = 0.71, *p* = 0.03), and psychoticism (aRRR = 0.72, *p* = 0.04) subscales were more likely to disclose decreased than unchanged cigarette consumption levels.

**Table 1:** Multinomial regression of sociodemographic and psychometric variables against perceived changes of substance use during the pandemic.

**Variables**	**Perceived alcohol consumption change^a^**				**Perceived cigarette consumption change^a^**			
	**Decreased**		**Increased**		**Decreased**		**Increased**	
	**aRRR**^**c**^	**95% CI**	**aRRR**^**c**^	**95% CI**	**aRRR**^**c**^	**95% CI**	**aRRR**^**c**^	**95% CI**
Male^b^	0.94	0.56–1.72	1.04	0.63–2.24	0.64	0.30–1.36	2.70^**^	1.32–5.50
Age	0.99	0.94–1.04	1.04	0.99–1.09	0.99	0.96–1.01	1.01	0.98–1.05
**Education**								
University Graduates	0.64	0.31–1.33	2.38^*^	1.02–5.58	1.23	0.83–1.81	1.44	0.89–2.32
Diploma	1.96	0.67–5.71	3.15	0.94–10.48	0.69	0.38–1.27	0.83	0.41–1.69
Up to Senior High	Ref				Ref			
**Occupation**								
Professionals	0.48	0.16–1.44	0.48	0.10–2.29	0.54	0.12–2.47	2.93	0.41–21.03
Office Workers	1.49	0.56–3.98	1.54	0.37–6.34	0.58	0.19–1.77	2.42	0.42–13.90
Civil Servants	0.52	0.11–2.58	0.48	0.07–3.48	0.27	0.05–1.40	2.90	0.37–22.49
Unemployed	1.06	0.13–8.81	1.67	0.20–14.03	-	-	-	-
Students	Ref				Ref			
**Marital status**								
Single	1.15	1.27–3.59	0.31^***^	0.16–0.59	1.03	0.67–1.58	1.69^*^	1.04–2.75
Married/Divorced	Ref				Ref			
**Income**								
Low	0.59	0.16–2.18	1.61	0.41–6.29	1.52	0.63–3.69	2.69	0.84–8.61
Lower-Middle	0.87	0.39–1.98	2.39	0.99–5.79	0.6	0.33–1.10	1.73	0.80–3.75
Upper-Middle	1.24	0.61–2.50	1.45	0.66–3.17	0.78	0.44–1.38	1.89	0.91–3.91
High	Ref				Ref			
**Province**								
Implement PSBB	2.14^**^	1.27–3.59	1.07	0.62–1.87	0.68^*^	0.48–0.96	1.14	0.77–1.69
Not Implement PSBB	Ref				Ref			
**Physical distancing**								
Practice	0.87	0.50–1.53	1.03	0.58–1.84	0.95	0.67–1.33	1.14	0.77–1.68
Do Not Practice	Ref				Ref			
**COVID-19 confirmed/suspected cases within household**								
Present	1.82	0.61–5.46	1.53	0.46–5.08	0.86	0.36–2.05	0.55	0.17–1.77
Absent	Ref				Ref			
AUDIT	1.11^***^	1.05–1.18	1.05	0.99–1.12	-			
CDS	-				0.95^***^	0.94–0.97	1.06^***^	1.04–1.08
PSQI	1.07	0.97–1.18	1.11^*^	1.01–1.23	1.02	0.96–1.09	1.03	0.96–1.11
**SCL-90**								
GSI	0.99	0.63–1.57	0.8	0.47–1.36	1.32	0.97–1.79	0.99	0.67–1.46
Depression	0.95	0.60–1.51	1.18	0.69–2.01	0.75	0.55–1.03	0.99	0.67–1.48
Anxiety	1.00	0.62–1.62	1.36	0.78–2.39	0.83	0.61–1.14	0.99	0.67–1.48
Obsessive-Compulsive	1	0.60–1.66	1.28	0.71–2.30	0.76	0.54–1.07	1	0.65–1.52
Phobic Anxiety	1.02	0.64–1.63	1.19	0.70–2.05	0.70^*^	0.51–0.96	1	0.67–1.49
Somatization	1.04	0.66–1.65	1.22	0.72–2.07	0.78	0.57–1.14	1.02	0.69–1.51
Interpersonal Sensitivity	1.14	0.70–1.85	1.48	0.84–2.61	0.81	0.58–1.14	1.02	0.67–1.56
Hostility	1.01	0.63–1.64	1.27	0.72–2.23	0.71^*^	0.52–0.97	1.08	0.73–1.60
Paranoid Ideation	1.01	0.63–1.61	1.07	0.63–1.83	0.74	0.54–1.03	1.02	0.68–1.52
Psychoticism	0.91	0.57–1.45	1.26	0.73–2.17	0.72^*^	0.52–0.99	0.98	0.66–1.47
Additional	0.99	0.61–1.61	1.22	0.70–2.12	0.76	0.55–1.05	1.02	0.68–1.52
−2 Log likelihood	814.7, *p* ≤ 0.001				1772.8, *p* ≤ 0.001			
Nagelkerke R^2^	0.27				0.19			

a Reference category is stable (alcohol/cigarette) change;

b Female is the reference;

c aRRR, adjusted relative risk ratio;

* p < 0.05;

** p ≤ 0.01;

*** *p ≤ 0.001*.

## Discussion

Overall, less than a tenth of our sample disclosed consuming alcohol during the pandemic, while one-fifth reported daily cigarette smoking over the same period. Traditionally, Indonesia's alcohol consumption has been documented to be lower than that of other countries. In this study as well, most of the respondents reported that they did not consume alcohol but there was an observable increase compared to a survey by the National Board of Narcotics in 2018 that found only around 3.0% of the total Indonesian adult population consumed alcoholic beverages in the past year (24). The generally low alcoholic consumption might be attributed to the fact that the majority of the Indonesian population practices Islam as a religion and considers the consumption of alcohol to be immoral (25). In comparison, the prevalence of 20.3% daily cigarette smoking during the pandemic was relatively maintained compared to the daily tobacco smoking rate, 24.3%, in 2018 (9). In support of this, cigarettes and tobacco are not forbidden by Islamic teachings, leading to its wider consumption by the public.

This study demonstrated the changing patterns of usage of two substances among Indonesian adults during the PSBB period in Indonesia, from April to July 2020. Generally, most respondents reported unchanged intensity of consumption, and both cigarette and alcohol usage had a larger proportion of respondents reporting decreased consumption than increased. The changes of alcohol consumption observed in this study is similar to Australian and Polish data, with the highest proportion being stable alcohol drinking, followed by reduced usage, and the least was increased consumption (26, 27). Furthermore, we found that physical distancing did not account for any fluctuation in alcohol usage, although the implementation of the PSBB regulation within provinces did correlate with decreased alcohol consumption. The PSBB introduced much wider policies apart from social and mobility limitations to include stores' closures, which would have impacted alcohol products' availability. During this period, nearly half of the affected Indonesians reported minimizing going out of their homes, over 80% believed they were susceptible to COVID-19 (28), and the government had been debunking hoaxes on alcohol ingestion as a coronavirus prevention (29). Perplexingly, a higher AUDIT score was associated with reduced alcohol than maintenance; although, over 80% of the respondents were consuming alcohol reasonably, which could then suggest that social limitations such as diminished availability and accessibility had stronger suppressive effects. It is necessary to keep in mind that alcohol consumption comprises a spectrum from social drinking to pathological drinking behavior (30). In contrast to some studies (31, 32) in the Western hemisphere where alcohol was stockpiled during the lockdown, it is unlikely that alcohol hoarding occurred in Indonesia due to the scarcity of alcohol and limited availability due to the pandemic. This could be a reason for the reduced alcohol consumption among the respondents in this study, as shown by the low AUDIT score on average. The perceived decrease in alcohol consumption might be in line with the WHO statement that the current situation is a unique opportunity to reduce drinking considerably (33). Thus, obedience to limit physical contact, fear of COVID-19 infection, and low rates of alcohol dependence might be attributed as the reasons leading to decreased alcohol consumption in Indonesia.

In contrast to previous findings, being single was associated with lower risk of reporting increased alcohol than stable alcohol consumption in our study. Concordantly, some studies have suggested that social distancing and staying home during the COVID-19 pandemic could disrupt couples' and families' routines and lead to domestic violence escalation, ultimately resulting in additional marital distress (34, 35) and driving up alcohol consumption. In addition, marriage to a spouse with alcohol use disorder has been known to increase the risk for alcohol-related disorders (36). Moreover, attainment of university education is correlated with twice the risk of increased than stable alcohol drinking during the pandemic in Indonesia, although this did not seem to be influenced by financial affluence as no significant relationship was observed between income bands. Previously, it was shown that those with a college degree as opposed to those without demonstrated higher increases of at-risk drinking between adolescence and adulthood and the pattern was specific to alcohol and not tobacco or marijuana (37, 38). Among college students, research also noted positive correlation of alcohol consumption and higher subjective level of well-being and self-efficacy (39, 40). These could imply substance preference and unique demographic or academic characteristics influencing alcohol use among those able to attain a university degree in the face of stressors. Another probable consequence of this stressor was sleeping disturbance that was quite prevalent among the respondents. Poor sleep quality correlated significantly to increased alcohol use and this similar pattern was observed with internet addiction among Indonesian adults during the pandemic (41). Alcohol acutely acts as a sedative on non-alcoholic people, which will shorten sleep latency but perturb the rapid eye movement (REM) sleep cycle. Among alcoholics, studies documented that continued use and abuse results in tolerance to its sedative effects, and as such, they develop irregular sleep-wake cycles, deprived REM sleep duration, and daytime sleepiness (42). In turn, poor sleep quality is also suggested to trigger excessive alcohol use (43). Consequently, pervasive sleep disturbance had been recorded during the COVID-19 pandemic and lockdown period across the globe (44), which might intensify alcohol use in vulnerable individuals.

In this study, cigarettes were the more commonly used substance during the COVID-19 pandemic. However, approximately two-thirds more respondents reported a decrease in cigarette smoking than increase during the pandemic. Contradictorily, a Chinese study reported a slight increase (0.8%) in cigarette smoking prevalence during COVID-19 (45), while an Australian study indicated that cigarette smoking tended to decrease (26). On the other hand, another study described similar proportions of decreased and increased cigarette smoking (46) and a multinational study reported a nearly unchanged pattern in consumption of tobacco cigarettes (47). Globally, there were differences in the changes in cigarette consumption during the pandemic; however, we observed a higher degree of the population reporting regular consumption in Indonesia, with increased smoking least mentioned. Unlike alcohol, the tax for a cigarette in Indonesia is lower, 58.5%, (12) than the set global cigarette tax (70%) (48). Hence, cost-wise, a cigarette is much more accessible in Indonesia and sales of cigarettes might be less affected by the economic crisis, which partly explained the unchanged intensity of cigarette smoking in nearly half of the smokers and the non-significant relationship to household income seen in this study. Additionally, the gap between reduced and increased addictive substance consumption was more notable among smokers (32.3 vs. 20.1%) than among alcohol drinkers, which could be partly attributed to the intensive public education on the negative association between smoking and COVID-19 (49).

Expectedly, gender was significantly correlated with changes in cigarette consumption and our study sample indicated that males were nearly three times more likely than females to report increased smoking than stable cigarette consumption. To a certain degree, this could be attributed to the heightened male's, than female's, activation of the reward pathway from nicotine (50) and the oppressive societal and religious norms subjected upon women (51). Previous evidence suggested that stress response is a driving force of tobacco initiation and relapse in women (52). However, it might not principally affect current female smokers' tendency to modify their tobacco smoking since it provided lower rewarding sensation than for males (53). Furthermore, unmarried respondents were at least 50% more likely than married respondents to have increased than unchanged smoking during the pandemic. A previous study also reported a similar pattern of lower smoking prevalence in married households (54). Respondents who reported an increase in cigarette consumption during the pandemic had the highest scores on CDS-10, which signified heightened dependence. Higher CDS-10 scores were significantly linked to higher risk of reporting increased than stagnant cigarette usage but less likely to report decreased than unchanged cigarette smoking. Thus, indicating that respondents with advanced dependence require further assistance and incentives to reduce their smoking. Especially during this pandemic, cigarette smoking has been linked with the upregulation of angiotensin-converting enzyme-2 in lung cells (55, 56) and weakened immune system (57); it was also reported that patients with smoking history were associated with more progressed COVID-19 symptoms (58). Moreover, cigarette smoking has been linked to aggravating neuropsychiatric symptoms, systemic inflammation, coagulation, and other clinical symptoms in COVID-19 patients through nicotine and nicotinic-cholinergic system interaction (59, 60). The rising cigarette consumption in some respondents might be driven by factors other than psychopathologies, as neither significant difference in SCL-90 scores nor correlation in the regression analysis were seen. Decreased cigarette smoking was correlated with higher scores in phobic anxiety, interpersonal sensitivity, and psychoticism, which might be driven by lowered nicotine consumption (61). This interpretation remained limited within the context of current data and should be investigated in future studies.

This study had some limitations. First, the study only covered participants who could access this survey through the internet. Subjective bias could also have occurred as this survey used self-reported questionnaires. Previous national data on the substance use pattern before the COVID-19 pandemic were limited, restricting an in-depth analysis of the current situation. The current study also did not employ a random sampling approach due to the limited timeframe. This study could not identify if respondents had only initiated consuming the substances during the study period and determine the previous level of use (acceptable, dependent, or hazardous). History of remission, withdrawal, or relapse (if any) was not collected as well. The details of distinct types of alcohol (e.g., wine, beer, spirits, etc.) and cigarettes (e.g., combustible or e-cigarette) were not explored in this study. Further follow-up studies are required to assess the shortcomings of this study and the consequences of pandemic policies (e.g., physical distancing) on addiction in the long term. However, to the best of our knowledge, this is the first study to investigate the changes of substance use during the COVID-19 pandemic in Indonesia. This study could be used as a reference to modify the nationwide approach to the COVID-19 pandemic and addiction across prevention and intervention policies. Despite minimal personal mobilization and heightened fear toward COVID-19, (28) the majority of respondents could maintain their alcohol and cigarette consumptions, with a smaller proportion of respondents enhancing their consumption during the pandemic. Therefore, stakeholders should ensure the maintenance and intensification of psychiatric and addiction services during and after the pandemic.

## Data Availability Statement

The raw data supporting the conclusions of this article will be made available by the authors, without undue reservation.

## Ethics Statement

The studies involving human participants were reviewed and approved by Institutional Ethics Committee of Faculty of Medicine, Universitas Indonesia—Dr. Cipto Mangunkusumo General Hospital. The patients/participants provided their written informed consent to participate in this study.

## Author Contributions

EH and KS conceived, designed, and supervised the study. EH, KS, AL, HC, and LTS contributed data or analysis tools. EH, KS, AL, LTS, HC, A, LPS, and BM collected the data. EH, KS, AL, LTS, HC, and CS performed the data analysis. EH, KS, AL, LTS, HC, A, LPS, and BM wrote the manuscript. KS and CS secured funding for the study. All authors contributed to the article and approved the submitted version.

## Conflict of Interest

The authors declare that the research was conducted in the absence of any commercial or financial relationships that could be construed as a potential conflict of interest.
