# Supplementary material for: Alcohol- and Cigarette-Use Related Behaviors During Quarantine and Physical Distancing Amid COVID-19 in Indonesia
Source: Front Psychiatry. 2021 Feb 2;12:622917. doi: 10.3389/fpsyt.2021.622917 (PMC7884457; doi:10.3389/fpsyt.2021.622917)
Supplement: Supplementary file 1 [file Data_Sheet_1.docx]

Supplementary Material

# Supplementary Figures and Tables

Table S1. Sociodemographic variables of respondents according to perceived alcohol and cigarette consumption changes during the pandemic

| **Variables** | **Total (N)** | **Perceived Alcohol Consumption Change** | | | **χ2** | **Total (N)** | **Perceived Cigarette Consumption Change** | | | **χ2** |
| --- | --- | --- | --- | --- | --- | --- | --- | --- | --- | --- |
|  |  | **No Changes (N= 194)** | **Reduced (N= 130)** | **Increased (N= 112)** |  |  | **No Changes (N= 443)** | **Reduced (N= 301)** | **Increased (N= 187)** |  |
| **Age** |  | | | | | | | | | |
| ≤ 40 years old | 407 | 184 (45.2%) | 126 (31.0%) | 97 (23.8%) | 11.57** | 807 | 382 (47.3%) | 266 (33.0%) | 159 (19.7%) | 1.27 |
| > 40 years old | 29 | 10 (34.5%) | 4 (13.8%) | 15 (51.7%) |  | 124 | 61 (49.2%) | 35 (28.2%) | 28 (22.6%) |  |
| **Sex** |  | | | | | | | | | |
| Male | 263 | 111 (42.2%) | 81 (30.8%) | 71 (27.0%) | 1.44 | 870 | 421 (48.4%) | 286 (32.9%) | 163 (18.7%) | 15.08*** |
| Female | 173 | 83 (48.0%) | 49 (28.3%) | 41 (23.7%) |  | 61 | 22 (36.1%) | 15 (24.6%) | 24 (39.3%) |  |
| **Education** |  | | | | | | | | | |
| Up to Senior High | 63 | 28 (44.4%) | 23 (36.5%) | 12 (19.0%) | 4.85 | 228 | 114 (50.0%) | 77 (33.8%) | 37 (16.2%) | 8.79 |
| Diploma | 34 | 11 (32.4%) | 13 (38.2%) | 10 (29.4%) |  | 96 | 56 (58.3%) | 23 (24.0%) | 17 (17.7%) |  |
| University Graduates | 339 | 155 (45.7%) | 94 (27.7%) | 90 (26.5%) |  | 607 | 273 (45.0%) | 201 (33.1%) | 133 (21.9%) |  |
| **Occupation** |  | | | | | | | | | |
| Students | 28 | 14 (50.0%) | 11 (39.3%) | 3 (10.7%) | 22.25** | 17 | 7 (41.1%) | 8 (47.1%) | 2 (11.8%) | 18.07* |
| Professionals | 67 | 43 (64.2%) | 15 (22.4%) | 9 (13.4%) |  | 24 | 10 (41.7%) | 6 (25.0%) | 8 (33.3%) |  |
| Office Workers/ | 314 | 123 (39.2%) | 98 (31.2%) | 93 (29.6%) |  | 859 | 414 (48.1%) | 280 (32.6%) | 165 (19.3%) |  |
| Civil Servants | 18 | 11 (61.1%) | 4 (22.2%) | 3 (16.7%) |  | 22 | 12 (54.5%) | 4 (18.2%) | 6 (27.3%) |  |
| Currently Unemployed | 9 | 3 (33.3%) | 2 (22.2%) | 4 (44.4%) |  | 8 | 0 | 3 (37.5%) | 5 (62.5%) |  |
| **Marital Status** |  | | | | | | | | | |
| Single | 189 | 67 (35.4%) | 46 (24.3%) | 76 (40.2%) | 36.89*** | 688 | 340 (49.4%) | 224 (32.6%) | 124 (18.0%) | 7.49* |
| Married | 247 | 127 (51.4%) | 84 (34.0%) | 36 (14.6%) |  | 243 | 103 (42.4%) | 77 (31.7%) | 63 (25.9%) |  |
| **Monthly Income** |  | | | | | | | | | |
| < Rp 1,300,000 (Low) | 22 | 12 (54.5%) | 5 (22.7%) | 5 (22.7%) | 5.52 | 44 | 14 (31.8%) | 22 (50.0%) | 8 (18.2%) | 10.6 |
| Rp 1,301,000 – 5,000,000 (Lower-Middle) | 122 | 56 (45.9%) | 29 (23.8%) | 37 (30.3%) |  | 355 | 182 (51.3%) | 103 (29.0%) | 70 (19.7%) |  |
| Rp 5,001,000 – 15,000,000 (Upper-Middle) | 214 | 90 (42.1%) | 73 (34.1%) | 51 (23.8%) |  | 446 | 207 (46.4%) | 144 (32.3%) | 95 (21.3%) |  |
| > Rp 15,000,000 (High) | 78 | 36 (46.2%) | 23 (29.5%) | 19 (24.4%) |  | 86 | 40 (46.5%) | 32 (37.2%) | 14 (16.3%) |  |
| **PSBB^a^** |  |  |  |  |  |  |  |  |  |  |
| Yes | 191 | 81 (42.4%) | 68 (35.6%) | 42 (22.0%) | 5.96* | 288 | 141 (49.0%) | 81 (28.1%) | 66 (22.9%) | 4.11 |
| No | 245 | 113 (46.1%) | 62 (25.3%) | 70 (28.6%) |  | 643 | 302 (47.0%) | 220 (34.2%) | 121 (18.8%) |  |
| **Physical distancing** |  | | | | | | | | | |
| Yes | 322 | 143 (44.4%) | 99 (30.7%) | 80 (24.8%) | 4.57 | 658 | 315 (37.9%) | 214 (32.5%) | 129 (19.6%) | 0.32 |
| No | 114 | 51 (44.7%) | 31 (27.2%) | 32 (28.1%) |  | 273 | 128 (46.9%) | 87 (31.9%) | 58 (21.2%) |  |
| **Suspected/Confirmed COVID-19 Cases within Household** |  | | | | | | | | | |
| Yes | 23 | 8 (34.8%) | 8 (34.8%) | 7 (30.4%) | 0.93 | 29 | 15 (31.7%) | 10 (34.5%) | 4 (13.8%) | 0.74 |
| No | 413 | 186 (45.0%) | 122 (29.5%) | 105 (25.5%) |  | 902 | 428 (47.5%) | 291 (32.3%) | 183 (20.3%) |  |

Notes: ^a^ Large-scale social restrictions; * p<0.05; ** p≤ 0.01; ***p≤ 0.001

Table S2. Descriptive analysis of study variables across perceived changes in alcohol and cigarette consumption during the pandemic

| **Variables** | **Perceived Alcohol Consumption Change** | | | **F-ratio** | **Post-hoc** | **Perceived Cigarette Consumption Change** | | | **F-ratio** | **Post-hoc** |
| --- | --- | --- | --- | --- | --- | --- | --- | --- | --- | --- |
|  | **Stable (1)** | **Decrease (2)** | **Increase (3)** |  |  | **Stable (1)** | **Decrease (2)** | **Increase (3)** |  |  |
| **Age** | 29.67±6.21 | 29.42±5.83 | 32.85±6.78 | 10.16*** | 3>1 3>2 | 33.72±7.48 | 32.97±6.70 | 32.98±7.17 | 1.20 |  |
| **AUDIT** | 2.82±3.69 | 4.85±4.56 | 3.20±5.84 | 7.99*** | 1>2 | - | | |  |  |
| **CDS** | - | | |  |  | 24.89±8.86 | 22.01±7.72 | 28.72±9.03 | 35.72*** | 3>2>1 |
| **PSQI** | 5.36±3.04 | 5.61±3.15 | 5.87±3.63 | 0.88 |  | 5.16±3.07 | 5.55±3.11 | 5.46±3.05 | 1.61 |  |
| **SCL** |  | | | | |  | | | | |
| **GSI** | 62.04±70.20 | 55.37±62.34 | 74.91±71.79 | 2.52 |  | 29.28±44.08 | 38.34±53.61 | 31.05±38.60 | 3.54* | 2>1 |
| **Depression** | 10.04±11.87 | 9.02±10.61 | 13.06±12.83 | 3.84* | 3>2 | 4.32±6.80 | 5.68±8.84 | 4.52±6.43 | 3.15* | 2>1 |
| **Anxiety** | 6.08±8.22 | 4.97±6.89 | 7.13±8.07 | 2.32 |  | 2.51±4.73 | 3.60±6.08 | 2.64±3.99 | 4.45* | 2>1 |
| **Obsessive-Compulsive** | 7.91±8.50 | 7.43±7.64 | 9.66±8.51 | 2.44 |  | 3.77±5.57 | 4.92±6.45 | 3.90±4.86 | 3.82* | 2>1 |
| **Phobic Anxiety** | 3.71±5.42 | 3.47±4.99 | 4.06±5.54 | 0.38 |  | 2.10±3.59 | 2.39±4.10 | 2.07±3.06 | 0.66 |  |
| **Somatization** | 7.25±9.00 | 5.92±8.14 | 7.79±8.46 | 1.57 |  | 3.78±6.07 | 5.13±7.59 | 4.03±5.55 | 4.02* | 2>1 |
| **Interpersonal Sensitivity** | 7.42±8.41 | 6.85±7.55 | 8.90±9.12 | 1.92 |  | 3.10±4.93 | 4.18±6.18 | 3.42±4.61 | 3.72* | 2>1 |
| **Hostility** | 2.95±4.10 | 2.69±3.76 | 3.82±4.85 | 2.38 |  | 1.51±2.79 | 1.87±3.19 | 1.72±2.66 | 1.38 |  |
| **Paranoid Ideation** | 4.20±5.57 | 3.74±4.85 | 5.02±6.01 | 1.67 |  | 2.00±3.62 | 2.53±3.79 | 2.23±3.39 | 1.93 |  |
| **Psychoticism** | 5.97±7.81 | 5.18±7.11 | 6.95±8.20 | 1.57 |  | 2.69±3.09 | 3.49±5.88 | 2.84±4.39 | 2.16 |  |
| **Additional** | 5.51±5.91 | 5.20±5.37 | 7.34±6.00 | 4.88** | 3>1 3>2 | 3.08±4.23 | 3.95±4.97 | 3.23±3.97 | 3.57* | 2>1 |

Notes: * p<0.05; ** p≤ 0.01; ***p≤ 0.001
